# A role for topoisomerase II*α* in the formation of radiation-induced chromatid breaks

**DOI:** 10.1038/sj.bjc.6604514

**Published:** 2008-07-29

**Authors:** S Y A Terry, A C Riches, P E Bryant

**Affiliations:** 1 Bute Medical School, Bute Medical Buildings, University of St Andrews, St Andrews KY16 9TS, Scotland, UK

**Keywords:** topoisomerase II*α*, cancer susceptibility, HL60, chromosome damage, radiation sensitivity

## Abstract

Chromatid breaks in cells exposed to low dose irradiation are thought to be initiated by DNA double-strand breaks (DSB), and the frequency of chromatid breaks has been shown to increase in DSB rejoining deficient cells. However, the underlying causes of the wide variation in frequencies of G2 chromatid breaks (or chromatid ‘radiosensitivity’) in irradiated T-lymphocytes from different normal individuals and cancer cases are as yet unclear. Here we report evidence that topoisomerase II*α* expression level is a factor determining chromatid radiosensitivity. We have exposed the promyelocytic leukaemic cell line (HL60) and two derived variant cell lines (MX1 and MX2) that have acquired resistance to mitoxantrone and low expression of topoisomerase II *α*, to low doses of *γ*-radiation and scored the induced chromatid breaks. Chromatid break frequencies were found to be significantly lower in the variant cell lines, compared with their parental HL60 cell line. Rejoining of DSB in the variant cell lines was similar to that in the parental HL60 strain. Our results indicate the indirect involvement of topoisomerase II*α* in the formation of radiation-induced chromatid breaks from DSB, and suggest topoisomerase II*α* as a possible factor in the inter-individual variation in chromatid radiosensitivity.

Human response to low doses of ionising radiation shows a wide variation as exemplified by the different frequencies of radiation-induced chromatid breaks observed in metaphase chromosomes in phytohaemagglutinin-stimulated peripheral blood T-lymphocytes (PBL) from different normal individuals and sporadic cancer cases, and elevated frequencies of such chromatid breaks have been linked to cancer susceptibility (Scott *et al*, 1994, 1996; Roberts *et al*, 1999; Baria *et al*, 2001, 2002; Buchholz and Wu, 2001; Riches *et al*, 2001; Papworth *et al*, 2001; Smart *et al*, 2003; Baeyens *et al*, 2002, 2005). Using a short time interval (1–2 h) between radiation exposure and sampling, the metaphase cells (blocked with colcemid) collected are those that were in the G2-phase of the cell cycle at the time of exposure and show frequent chromatid breaks (discontinuities or terminal deletions) that have been shown to be induced as a linear function of radiation dose (Bryant, 1998).

Cell-cycle arrest is a factor that could possibly influence observed frequencies of chromatid breaks, and at least one study of human tumour cell lines *in vitro* (Schwartz *et al*, 1996) reports an inverse relationship between mitotic inhibition and chromatid break frequency. Also, using premature chromosome condensation (PCC) in G2-phase cells with phosphatase inhibitor calyculin, G2 cells were distinguished from normal mitotic metaphases by their split centromeres and by the loss of centromeric constriction (Terzoudi *et al*, 2005). Using this feature of calyculin-induced PCC to distinguish G2 cells from mitotic metaphase cells, these authors reported that cells from individuals with ataxia telangiectasia (A–T), a homozygous autosomal syndrome characterized by partial abrogation of radiation-induced G2-phase cell-cycle delay and high frequencies of chromatid breaks when compared with normals, report no difference in chromatid break frequencies between A-T cells and those from normal individuals. However, other studies using PBL from sporadic breast cancer patients and normal controls show only a very weak or no correlation between cell-cycle delay and chromatid break frequencies at the low radiation dose normally used in the ‘G2 assay’ of chromatid breaks (Scott *et al*, 2003; Pretazzoli *et al*, 2000) suggesting that although cell-cycle checkpoint delay may have some influence on G2 chromatid break scores in stimulated PBL, it is not a major determinant of chromatid break frequency in sporadic cancer cases, especially if the test radiation dose is kept to 0.4–0.5 Gy where the effect on cell-cycle delay is minimal.

The classical (and probably the prevailing current) view of radiation-induced chromosomal aberrations avers that chromatid breaks simply represent expansions of DNA double-strand breaks (DSB). However, this view has to be questioned in the light of reports showing a lack of correspondence between the kinetics of the disappearance of chromatid breaks with time and those of DSB rejoining following radiation exposure (Bryant *et al*, 2004). Based on such observations we proposed an alternative hypothesis, namely that chromatid breaks are indirectly formed by the initial presence of a DSB, and that the formation could involve the decatenation of chromatids by the DNA processing enzyme topoiosmerase II*α* (Bryant, 2007).

Topoisomerase II is a nuclear enzyme that is known to be involved in many cellular processes including replication, transcription, sister chromatid decatenation, and as such is crucial for cell survival (Austin and Marsh, 1998). Currently, many antineoplastic drugs such as etoposide and amsacrine are being used clinically as they stabilise the ternary complex and block the normal religation of the cleaved DNA by the enzyme, resulting in a high level of DSB causing cells to undergo apoptosis, or formation of translocations (Felix *et al*, 2006). Topoisomerase II is present in two isoforms (*α* and *β*). We think it likely that the *α*- rather than the *β*-isoform is involved in the formation of chromatid breaks as the expression of topoisomerase II*α* is variable during the cell cycle, peaking during the G2-phase where it is involved in chromatid decatenation. In contrast the *β*-isoform remains constant throughout the cell cycle (Woessner *et al*, 1991). Our hypothesis is that the presence of a DSB within a looped chromatin domain chemically or physically alters the loop structure in such a way as to enhance the error-proneness of topoisomerase II*α* during chromatid decatenation in G2-phase, resulting in either complete loop excision or misjoining leading to inversion of the looped domain adjacent to the chromatid break (Bryant, 2004, 2007). Another possibility is that a DSB, locally associated with other types of DNA damage, for example, abasic sites (Sutherland *et al*, 2000) could cause error-proneness of topoisomerase II*α*, causing illegitimate rearrangements within a damaged chromatin domain of a radiation-exposed cell. Error-proneness of topoisomerase II*α* has been shown to be triggered by exposure to reactive oxygen species (Li *et al*, 1999) and could also be engendered by endogenous or radiation-induced abasic sites, which are known to act as potent topoiosmerase II*α* poisons (Kingma *et al*, 1995).

Here we report on experiments to test the hypothesis that topoisomerase II*α* is involved in the formation of chromatid breaks in radiation-exposed G2 cells.

## Materials and methods

### Cell culture

Promyelocytic leukaemic parental HL60 and mitoxantrone-resistant variants MX1 and MX2 cell lines (ATCC, Manassas, VA, USA) were grown in RPMI-1640 (Gibco, Paisley, UK) medium containing 10% foetal calf serum (Globefarm Lrd, Cranleigh, UK), 2 mM L-glutamine (Invitrogen Life Technologies, Paisley, UK), 50 *μ*g ml^−1^ streptomycin (Invitrogen) and 50 U ml^−1^ penicillin (Invitrogen) at 37°C and in 5% CO_2_.

### Lysate preparation and protein quantification

Exponentially growing cells were washed twice in phosphate-buffered saline (PBS) (Gibco) and resuspended in a 14 *μ*l per 10^6^ cells sample reducing buffer (2%SDS (BDH Biochemical, Poole, UK), 100 mM DTT, 10% glycerol (BDH), 60 mM Tris pH 6.8. (BDH), 0.1% bromophenol blue). Protein was then denatured for 5 min in boiling water and stored at −20°C.

The amount of protein was quantified using the Bio-Rad Laboratories Protein Assay in which known BSA (Promega Corporation, Southampton, UK) concentrations, namely 0–8 *μ*g ml^−1^, were used as reference values at a 595 nm wavelength (Hemel, Hempstead, UK).

### Immunoblotting

A total of 11 *μ*g of cell lysate was run on a 7% SDS polyacrylamide gel using a Bio-Rad PowerPac 300 (Bio-Rad Laboratories, Herts, UK). Proteins were transferred on to a nitrocellulose membrane (Watman, Schleicher & Schuell, Dassel, Germany) at 100 V for 1 h and checked with Ponceau red stain. Protein epitopes were blocked in 2% milk powder in PBS/Tween for 15 min. Membranes were further incubated for 2 h (or at 4°C overnight) in either rabbit anti-topoisomerase II*α* or mouse anti-*β*-actin (Abcam, Cambridge, UK) at 1 : 20 000 and 1 : 5000, respectively, in PBS/Tween-20. After being washed with PBS/ Tween, the membranes were incubated in either anti-mouse or anti-rabbit antibody both of which were horseradish peroxidase-conjugated (Pierce Biotechnology, Rockford, IL, USA) at 1 : 100 000 dilution. Membranes were then washed twice in PBS/Tween and the secondary antibodies were detected by enhanced chemiluminescence (Millipore, Watford, UK). The amount of protein in the samples was estimated using image analysis.

### Mitotic index

Exponentially growing HL60, MX1 and MX2 cells (1 × 10^6^) were incubated in various concentrations of amsacrine hydrochloride (mAMSA, Sigma, Poole, UK; stock in 30% ethanol), ranging from 0 to 20 *μ*M for 30 min at 37°C. Cells were centrifuged and washed in the growth medium before being resuspended and incubated in the growth medium with 0.15 *μ*g ml^−1^ colcemid (Gibco) for 2 h at 37°C before being washed in the medium by centrifugation. Cells were fixed in 70% ethanol for 30 min on ice. After centrifugation, cells were permeabilised and stained for phospho-histone H3 according to the manufacturer's protocol (Cell Signalling Technology, Danvers, MA, USA). Cells were also incubated for 30 min at 37°C in 1 mg ml^−1^ ribonuclease A in 0.5% donkey serum in PBS, centrifuged and resuspended in a solution of 2 *μ*g ml^−1^ propidium iodide in PBS for 30 min at room temperature before being washed in PBS and analysed by FACScan (Becton-Dickinson BioSciences, Oxford, UK) with CellQuest software (San Jose, CA, USA).

### Chromatid break analysis

Exponentially growing HL60, MX1 and MX2 cells were irradiated with a dose of 0.4 Gy. and with unirradiated controls incubated for 30 min before the addition of 0.1 *μ*g colcemid for 1.5 h. Cells were then centrifuged and resuspended in hypotonic solution (75 mM KCl) for 7 min at 37°C. Cells were fixed three times in 3 : 1 methanol : acetic acid (BDH). Metaphase spreads were prepared using a humidity control cabinet (Hanabi, AO Science Technologies, Chiba, Japan). Slides were stained with 10% Giemsa (BDH) in Gurr's buffer (BDH) for 10 min. Hundred metaphases were examined for chromatid breaks using oil immersion ( × 100) optics (Zeiss Axioplan 2, Welwyn Garden City, Hertfordshire, UK). Chromatid breaks were defined as any chromatid discontinuity (Bryant *et al*, 2002).

### DNA double-strand break measurements

Cells were passaged at 2 × 10^5^ per ml in RPMI medium and incubated for 2 days. 6 × 10^5^ cells were transferred to 1.5 ml Eppendorf tubes and cooled on ice for 30 min prior to irradiation. Tubes were irradiated on ice with 40 Gy of *γ*-rays (IBL437C; CIS UK Bio-International, High Wycombe, UK). The dose rate was approximately 3.5Gy per min. Unirradiated controls and time zero samples were held on ice while the other samples were transferred to a water-bath running at 37°C for 15 min to 3 h. Following irradiation and incubation, all samples were returned to ice and then centrifuged at 1°C. The medium was aspirated and cell pellets resuspended in 160 *μ*l of 0.8% low melting point agarose (Sigma) in PBS at 37°C. Eighty microlitres was transferred to each of the two gel plug moulds (BioRad Laboratories Ltd., Hemel Hemstead, UK) and placed on ice for approximately 5 min to set. Plugs containing cells were extruded into 1 ml of ice-cold lysis solution (0.4 M EDTA, 2% sodium N-lauryl sarcosine, 1 mg ml^−1^ proteinase K, pH 8.0) in Eppendorf tubes for 30 min. Tubes were incubated overnight (18 h) at 37^o^C. A 200 ml 0.8 % agarose gel (DNA Sub-cell, BioRad Laboratories, Hemel Hemstead, UK) was prepared in 0.5 Tris acetate EDTA buffer containing 1 *μ*g per ml ethidium bromide. The plugs were recovered from the lysis solution and placed in comb wells in a 200 ml 0.8% Ultrapure agarose (Life Technology Ltd., Paisley, UK) gel in 0.5 TBE and containing ethidium bromide (0.5 *μ*g ml^−1^) in a BioRad Sub-Cell horizontal electrophoresis apparatus. Wells were sealed using 0.8% LMP agarose in PBS, and the gel was run in 0.5 TBE at 0.6 V cm^−1^ (6 mA, 8V; constant current) for 96 h.

The fraction of DNA released from the wells during electrophoresis was used as a measure of the induced double-strand breakage. DNA was quantified by ethidium bromide fluorescence, analysed using Syngene Genetools software (Syngene, Cambridge, UK). The mean fraction of DNA released (FDR) from each of the two wells per sample was determined by the following equation: 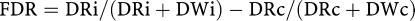 where:

DRi=DNA released (irradiated sample); DWi=DNA remaining in well (irradiated sample); DRc=DNA released (unirradiated control sample); DWc=DNA remaining in the well (control sample).

## Results

**Figure 1:**
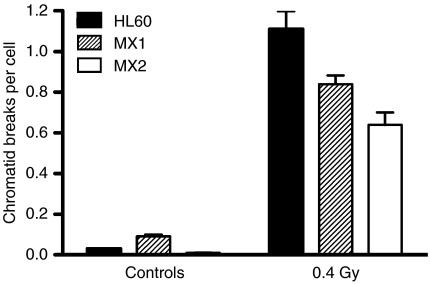
Frequency of chromatid breaks in control and irradiated HL60, MX1, and MX2 cells using the G2 assay.

**Figure 2:**
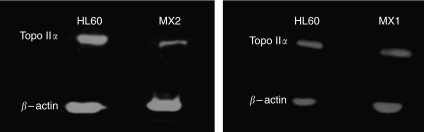
Western blots showing expression of topoisomerase II*α* in HL60, MX1 and MX2 cells.

**Figure 3:**
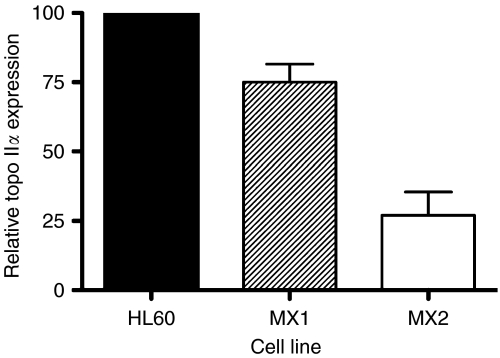
Relative topoisomerase II*α* expression level in HL60, MX1 and MX2 as estimated from western blots. Bars represent s.e.m. values from several experiments.

**Figure 4:**
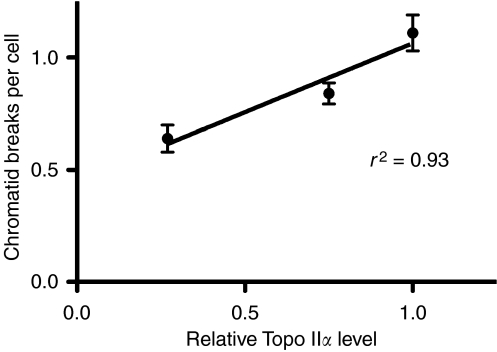
Chromatid break frequency as a function of relative topoisomerase II*α* level in HL60, MX1 and MX2 cells. Error bars represent standard errors of mean values.

**Figure 5:**
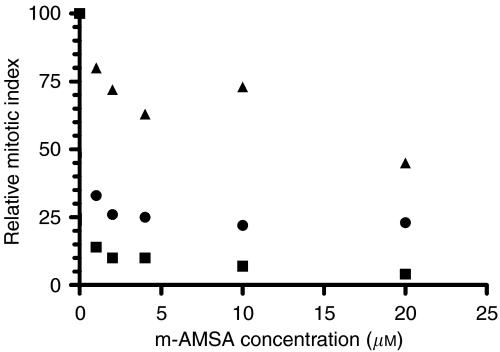
Mitotic index, measured by FACS in cells labelled with phospho-histone H3 labelled HL60 (squares), MX1 (circles) and MX2 (triangles) following treatment with mAMSA.

**Figure 6:**
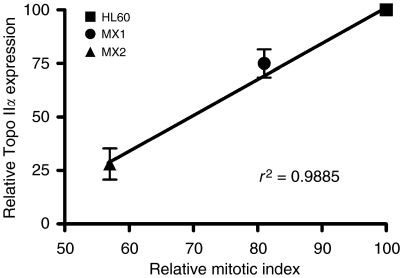
Relationship between relative topoisomerase II*α* and relative mitotic index (measured by FACS in cells labelled with phospho-histone H3) derived from data in Figure 5, at the highest mAMSA concentration used. Error bars represent s.e.m. values.

**Figure 7:**
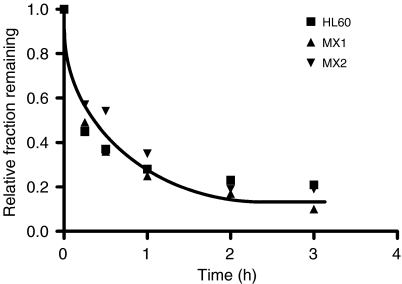
Rejoining of DSB following irradiation of HL60, MX1, and MX2 cell strains.

Figure 1 shows the results of two pooled experiments in which chromatid breaks were analysed in HL60 cells and variants MX1 and MX2 after exposure to *γ*-rays, sampling 1.5 h after using a 1 h colcemid block. The variant cell lines showed significantly lower chromatid break frequencies than the parental HL60 cell line (*P*=0.004 and 0.003 for MX1 and MX2 respectively). Western blots using topoisomerase II*α* antibody (Figure 2) indicates a reduced expression of topoisomerase II*α* in MX1 and MX2 compared with the parental HL60 line. Quantification of topoisomerase II*α* expression level using western blots, with *β*-actin as a control, shows a significantly lower expression of topoisomerase II*α* in the variant MX1 and MX2 strains (Figure 3). When chromatid break frequency is plotted against topoisomerase II*α* level (Figure 4) the data show a good correlation (*r*^2^=0.93). In a further test of topoisomerase II*α* expression in these cell lines (Figure 5), cells were exposed to mAMSA at varying concentrations and the effect on mitotic index was measured using phospho-histone H3 antibody in a FACS analysis. Both MX1 and MX2 were found to be significantly less sensitive to mAMSA than HL60. When the mitotic index (derived from those at the highest dose of mAMSA) was plotted against topoisomerase II*α* expression level derived from the western blot analysis shown in Figure 3, a good correlation was obtained *r*^2^=0.99 (Figure 6). Finally, the rejoining kinetics of DNA DSB was investigated in the three cell lines using constant-field agarose-gel electrophoresis (Figure 7). The rejoining kinetics in all three lines was found to be similar.

## Discussion

Our results demonstrate a close correlation between the frequency of radiation-induced chromatid breaks (or chromatid radiosensitivity) and the expression level of topoisomerase II*α* (Figure 4). MX1 and MX2 cells that have reduced expression of this enzyme show significantly lower chromatid break frequency in response to radiation. We have shown using western blot analysis that the expression level of topoisomerase II*α* in the variant mitoxantrone-resistant cell lines is significantly lower than in their HL60 parental cell line. Although the low topoisomerase II*α* expression in these variant cell lines had already been demonstrated by Harker *et al* (1991), it was necessary to ensure this was still the case as it was possible that the resistance of the cells to mitoxantrone could decrease over time, especially if cells were not kept under selection pressure by routine culture in mitoxantrone. This difference in topoisomerase II*α* expression is also reflected in the differential response of the three cell lines to mAMSA, a powerful topoisomerase II*α* poison leading to protein-associated DNA double-strand breaks (Davies *et al*, 1998). Reduced topoisomerase II*α* expression evidently leads to resistance to the inhibitory effect of mAMSA on mitosis (Figures 5 and 6).

As a check on the possibility that the differences seen in chromatid break frequencies in MX1 and MX2, compared with the HL60 cell line could be attributed to differences in DNA repair, we measured the rejoining of DSB. Our data (Figure 7) indicates no differences in DSB rejoining between the strains, and indicates that there is no involvement of topoisomerase II*α* in the rejoining of DSB. This finding is in agreement with the conclusions of Mandraju *et al* (2008) who show distinctly different roles for the two isoforms of topoisomerase II, namely that although topoisomerase II*α* upregulation is linked to enhancement of damage to DNA during treatment with H_2_O_2_ (possibly by relaxing and opening chromatin structure), it is only topoisomerase II*β* that is involved in DSB rejoining.

From these results we deduce that topoisomerase II*α* has an indirect or secondary role in the formation of chromatid breaks from DSB. We have previously proposed (Bryant, 2007) that topoisomerase II*α* might be rendered error-prone during chromatid decatenation by the presence of a DSB within a megabase looped domain in the chromatin. We suggested that such errors could result in misjoining of chromatin strands during the decatenation process leading to either loop excision or loop sequence inversion, which if remaining ‘incomplete’ (ie two of the chromatin strands are not joined) are manifested as chromatid breaks. It is known that some chromatid breaks involve interactions between chromatin strands of sister chromatids, that is, *inter*-chromatid rearrangements, and it has been proposed that these interactions could involve misjoining at the cross-over points of large looped domains (eg Harvey and Savage, 1997). Such rearrangements can be visualised using harlequin (fluorescence plus giemsa; FPG) staining, and result in the appearance of colour switches between chromatids at the break-points that occur at a frequency of approximately 16% (eg Harvey and Savage, 1997; Bryant, 1998). It is thought that the remainder results from intra-chromatid rearrangements, as described above. The reasons why a direct correlation between DSB and chromatid breaks is not probable have been discussed previously (Bryant 2004, 2007).

Perhaps one way in which topoisomerase II*α* might become error-prone, following exposure of cells to ionising radiation, is by the presence of DNA lesions such as abasic sites in proximity to DSB within looped chromatin structures. Such clustered damages, including abasic sites, have been demonstrated to be present after low radiation doses at frequencies similar to that of frank DSB (Sutherland *et al*, 2000). Abasic sites produced both endogenously by normal cellular metabolism, and as such are the most frequent lesion in normal mammalian cell DNA (occurring at some 10^4^ sites per cell per day), as well as being formed during excision repair and recombination following exposure to ionising radiation or reactive oxygen species.

Abasic sites have been shown to be potent topoisomerase II poisons, which are some 10 times more effective than etoposide (Kingma *et al*, 1995). Abasic sites, and more specifically apurinic sites, do not interfere with religation of topoisomerase II-induced DNA breaks, and although they can be quickly removed, if repair is faulty or a few sites are left behind, they can dramatically increase the formation of topoisomerase II-induced DSB (Kingma *et al*, 1995). Thus, following exposure to ionising radiation the combination of a DSB in a looped domain, simultaneously coupled with abasic-site poisoned topoisomerase II*α* could interact to form chromatid breaks.

The close correlation we have found between topoisomerase II*α* expression and the frequencies of chromatid breaks seen in the variant cell lines and HL60 parental line when exposed to low doses of ionising radiation leads us to suggest the possibility that both the inter-individual differences in chromatid radiosensitivity within groups of normal individuals, and the elevated chromatid radiosensitivity seen in cancer patients might result from differences in expression of topoisomerase II*α*.

In conclusion, our data show a strong correlation between radiation-induced chromatid break frequency and topoisomerase II*α* expression level, which we interpret as supporting our hypothesis of an indirect role for topoisomerase II*α* in the formation of chromatid breaks.
